# A Wearable Visually Impaired Assistive System Based on Semantic Vision SLAM for Grasping Operation

**DOI:** 10.3390/s24113593

**Published:** 2024-06-02

**Authors:** Fei Fei, Sifan Xian, Ruonan Yang, Changcheng Wu, Xiong Lu

**Affiliations:** College of Automation Engineering, Nanjing University of Aeronautics and Astronautics, Nanjing 211100, China; sifanxian@nuaa.edu.cn (S.X.); ruonanyang@nuaa.edu.cn (R.Y.); changchengwu@nuaa.edu.cn (C.W.); luxiong@nuaa.edu.cn (X.L.)

**Keywords:** visually impaired assistance, visual SLAM, human–computer interaction, semantic mapping

## Abstract

Because of the absence of visual perception, visually impaired individuals encounter various difficulties in their daily lives. This paper proposes a visual aid system designed specifically for visually impaired individuals, aiming to assist and guide them in grasping target objects within a tabletop environment. The system employs a visual perception module that incorporates a semantic visual SLAM algorithm, achieved through the fusion of ORB-SLAM2 and YOLO V5s, enabling the construction of a semantic map of the environment. In the human–machine cooperation module, a depth camera is integrated into a wearable device worn on the hand, while a vibration array feedback device conveys directional information of the target to visually impaired individuals for tactile interaction. To enhance the system’s versatility, a Dobot Magician manipulator is also employed to aid visually impaired individuals in grasping tasks. The performance of the semantic visual SLAM algorithm in terms of localization and semantic mapping was thoroughly tested. Additionally, several experiments were conducted to simulate visually impaired individuals’ interactions in grasping target objects, effectively verifying the feasibility and effectiveness of the proposed system. Overall, this system demonstrates its capability to assist and guide visually impaired individuals in perceiving and acquiring target objects.

## 1. Introduction

According to a report by the World Health Organization, at least 2.2 billion people worldwide have impaired vision due to myopia or hyperopia [1]. As over 80% of information is obtained through visual means for humans, the existence of visual impairments causes many difficulties for visually impaired individuals in acquiring information. Visually impaired individuals, as a special group among disabled people, face various problems in daily life, including poor independence and safety, employment difficulties, and other troubles [2,3,4].

To address the various difficulties faced by visually impaired individuals in their daily lives, assistive devices have been developed. Most existing assistive devices for visually impaired individuals rely on lasers, ultrasound, and other technologies to detect the location of obstacles. They can only provide tactile or auditory feedback to visually impaired individuals [5,6,7]. They cannot accurately identify and locate multiple obstacles at the same time. Some devices that can recognize target categories require additional ranging equipment but still cannot locate and identify multiple targets. Therefore, because of the limitations in various aspects, these assistive devices have not been widely accepted by visually impaired individuals as they do not provide significant convenience or friendly natural interaction experiences.

However, with the development of optical sensing technology, artificial intelligence technology, and human–computer interaction technology, new ideas and directions have been developed for the development of assistive devices for visually impaired individuals. Smart Specs is a smart glass that integrates a camera and computer, which can extract scene contour information and simplify colors. It projects the contour image onto the lens through a miniature projector to assist visually impaired people in perceiving scene information [8]. Takizawa et al. developed a depth camera with a cane to help visually impaired individuals find unoccupied seats. The system uses RANSAC-based depth data processing technology and face recognition algorithms to locate available seats [9]. Li et al. proposed a novel mobile-assisted navigation system called ISANA (Intelligent Situation Awareness and Navigation Aid) based on global vision, which can assist blind and visually impaired individuals with indoor autonomous navigation [10]. Jin et al. developed a wearable robot grasping assistant device (W-ROMA). W-ROMA is a novel hand-worn device that can assist visually impaired individuals in navigation and non-navigation tasks [11].

Semantic Visual SLAM integrates semantic information into traditional visual SLAM to achieve more advanced tasks. Object detection and semantic segmentation algorithms can be used to extract results instead of feature points, optimizing poses based on matching objects. In addition, a 3D semantic map containing scene semantic information (such as object size and category) can be constructed. Semantic Visual SLAM can provide more accurate positioning and navigation for visually impaired individuals by using semantic information algorithms to extract results instead of traditional feature points, combined with the SLAM algorithm to build a map and calculate the user’s current position and direction. Salas-Moreno et al. proposed the SLAM++ system, which can perform semantic mapping of indoor scenes. The system uses RGB-D visual sensors to identify known targets and uses them as landmarks in tracking and mapping [12]. McCormac et al. proposed a dense 3D semantic SLAM system called SemanticFusion, which combines the ElasticFusion framework with convolutional neural networks [13]. Niko et al. used SSD for object detection and integrated semantic information into ORB-SLAM2 by identifying objects’ semantic information on a point cloud map and using a point cloud segmentation algorithm to obtain an object-level semantic map [14]. Li et al. combined the LSD-SLAM framework with deep neural networks and used DeepLab-V2 for semantic segmentation. LSD-SLAM was used to estimate a camera’s pose and build a map, while CRF was used to optimize and build a semi-dense 3D semantic map [15].

**Figure 1:**
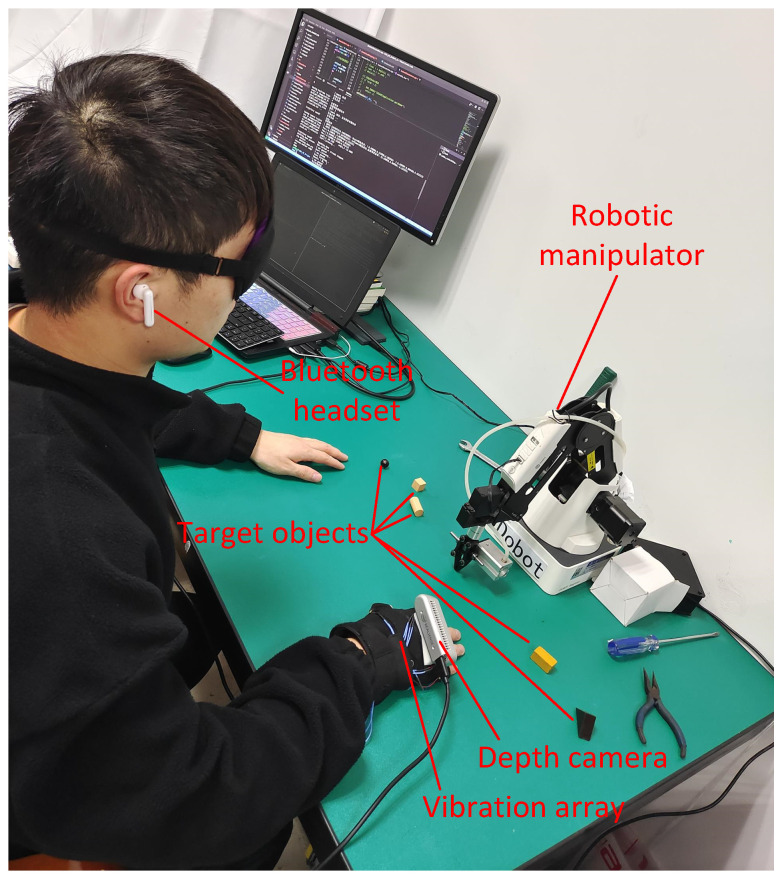
Demonstration of the visually impaired assistance system based on semantic visual SLAM.

In response to the challenges visually impaired individuals face in perceiving target objects, such as cups or tools, in their work and daily lives, this research paper presents an innovative assistive system for visually impaired individuals. By integrating wearable technology, human–computer interactions, and computer vision, a semantic visual SLAM-based assistive system is designed and implemented, as shown in Figure 1. This system aims to assist and guide visually impaired individuals in efficiently accessing target objects within desktop environments. This paper provides comprehensive explanations of the relevant theories, system architecture, and software and hardware design, as well as the development of semantic SLAM algorithms and experimental results about the assistive system for the visually impaired.

## 2. Wearable Visually Impaired Assistive System

The purpose of this paper is to design and implement a semantic visual SLAM-based visual impairment assistance system. The system aims to assist visually impaired individuals in obtaining information about target objects in a desktop scene by wearing the assistance device. The overall architecture design of the system will be completed based on the functional requirements, and both the software and hardware of each module will be designed according to their specific functions.

### 2.1. System Requirements

In order to assist visually impaired individuals in quickly and accurately obtaining target objects in a desktop environment, this paper proposes a hand-wearable visual assistive system. The system requirements are analyzed as follows:

(1) Construction of a sparse map and a semantic map.

The sparse map contains the geometric features of the desktop environment, which can assist visually impaired individuals in completing self-positioning tasks. However, the sparse map can only perceive whether there are objects in the environment; it cannot understand the semantic information of objects, such as their category and size, making it challenging to locate target objects. In contrast, the semantic map annotates the category and location of objects, so the system needs to establish a semantic map to enable more complex and intelligent interactive tasks.

(2) Relocalization based on a sparse map.

After constructing and saving the sparse map of the environment, the system can reload the map and use relocalization to determine its location in the map and perform continuous tracking. In addition, the system may lose track because of intense motion or object movement. When such external adverse effects disappear, relocalization can quickly restore the current pose.

(3) Accuracy and stability.

The system requires high positioning accuracy for target objects, i.e., the positioning accuracy of the target objects in the semantic map needs to be at the centimeter level. The camera coordinates on the device worn on the hand of a visually impaired individual are used to determine the placement of target objects. Therefore, the positioning accuracy of the system during operation also needs to reach the centimeter level. The system should be able to operate continuously and stably in dynamic or different environments without causing crashes or damage to hardware devices, ensuring the safety of visually impaired individuals.

(4) User interface.

The system should have a natural and friendly interaction process, using signals such as voice and vibration to exchange information between a visually impaired individual and the system, such as the position of the target object and the grasping result, to ensure that the system can understand the user’s interaction goals and that the visually impaired individual can know the current operating status of the system. Additionally, the device should be easy and safe to wear, without causing visually impaired individuals to experience complicated operational processes.

### 2.2. System Architecture

**Figure 2:**
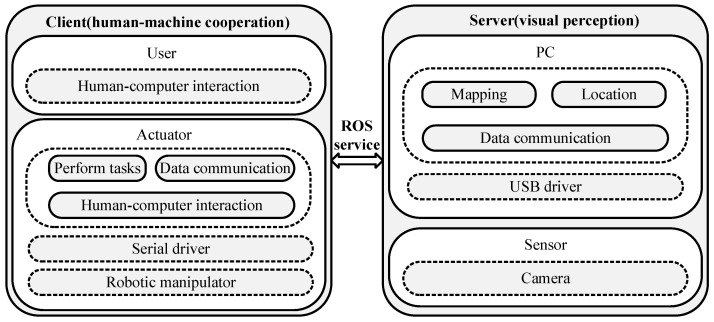
System architecture composed of the visual perception module as the server and the human–computer cooperation module as the client.

According to the above system requirements, the framework of the visually impaired assistance system under the ROS framework, as shown in Figure 2, mainly includes two modules as follows: the visual perception module and the human–machine cooperation module. The two parts are designed as a server–client system, and data exchange is performed through ROS service communication [16]. Among them, the visual perception module works as the server and is responsible for the positioning of the visually impaired person’s hand and the target object, as well as the map construction of the desktop environment. It can respond to client requests in real time, and after sending the positioning information to the human–machine cooperation module, it can assist or guide the visually impaired person to complete the operation of grasping target objects.

The visually impaired assistance system designed in this paper has two working modes as follows: the SLAM mode in unknown environments and the relocalization mode in known environments. The differences between them are explained as follows.

(1) SLAM mode in an unknown environment.

When the visually impaired person wears the assistance system in an unknown environment, the system will work in the SLAM mode. At this time, the human–machine cooperation module is not activated, and the visual perception module estimates its own pose in real time as the hand-held device moves while constructing a sparse map and a semantic map of the scene. If the command to complete the map construction is received, the system automatically saves the map and enters the relocalization mode.

(2) Relocalization mode in a known environment.

In this mode, the system initializes by loading the sparse map and semantic map created in the SLAM mode, and extracts map points and target object information from maps. When the visually impaired person’s assistance system moves and successfully relocates in the scene, the system obtains the positioning of the visually impaired person’s hand in the map and assists them in completing the operation of grasping the target object with interactive vocal commands.

### 2.3. Hardware Settings

The visual assistance system requires a camera to achieve scene perception. The RealSense D435i is an RGB-D camera produced by Intel Inc., which can simultaneously capture RGB and depth images. This camera has a maximum frame rate of 90FPS and a small size, making it suitable for wearing on the hands of visually impaired individuals. Therefore, it is used as the scene information acquisition device in the visual perception module.

**Figure 3:**
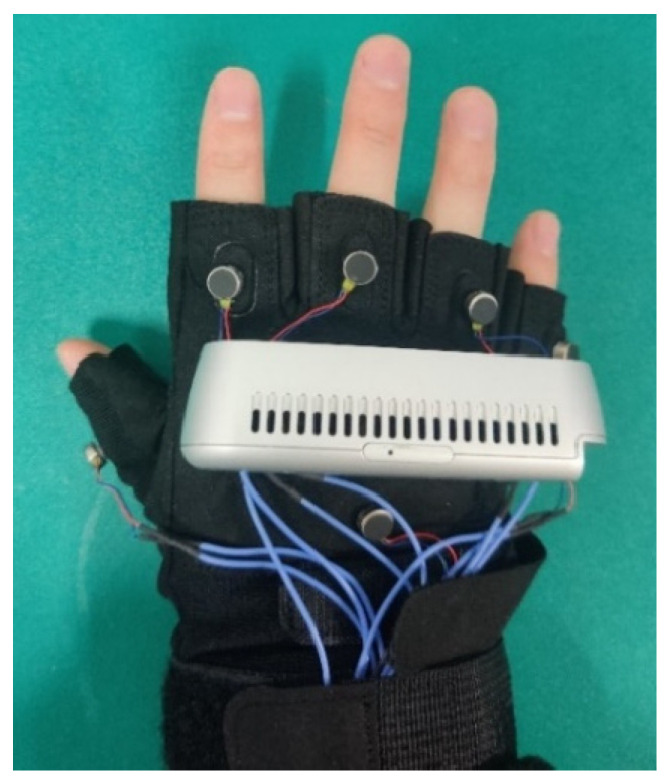
Wearable glove integrated with a depth camera and vibration motor array.

A vibration array feedback device that can convey direction and distance information is also developed, as shown in Figure 3. This feedback device can be used for both the assisted grasping mode and the active grasping mode. The assisted grasping mode involves controlling the robotic manipulator through voice interaction to assist visually impaired individuals in placing the target object in the specified position, directly in front of their hand, and then grasping the target object at that position. The active grasping mode guides visually impaired individuals to grasp the target object by combining voice and tactile interaction without a robotic manipulator.

As visually impaired individuals need to perform grasping actions with their hands, it is necessary to obtain the relative position between the target object and the hand coordinate system. Since the coordinates of the camera and the target object can be obtained through the semantic SLAM algorithm in the visual perception module, the camera is fixed to the visually impaired person’s hand by overlapping it with the hand, thereby obtaining the coordinate information of the target object relative to the visually impaired person’s hand. In order to fix the camera to the visually impaired person’s hand, this paper uses a half-fingered glove made of special nylon and super fiber as the basis for the wearable device. The camera of the visual perception end and the vibration array used for tactile interaction are fixed to the back of the glove with 3M high-viscosity double-sided tape, as shown in Figure 3.

The Dobot Magician robotic manipulator is a desktop-level intelligent robotic manipulator manufactured by Shenzhen Yuejiang Technology Inc. The working range of the Dobot robotic manipulator is semi-circular, with a radius of 320 mm, and a repeat positioning accuracy is 0.2 mm. On the 700mm × 700mm workbench faced by visually impaired individuals, the robotic manipulator can meet the requirements of grasping the target objects with an average radius of 20 mm, making it suitable as auxiliary equipment for the human–machine cooperation module. More details of the hardware settings are shown in Table 1.

### 2.4. Algorithm Description

The visual perception module needs to accomplish map construction, the positioning of target objects relative to the visually impaired user’s hand, and real-time communication with the client to respond to coordinate information requests. Traditional visual SLAM can achieve sparse or dense map construction of the environment and real-time pose estimation. However, using such maps can achieve functions, such as positioning and navigation, but cannot guide visually impaired individuals to obtain specific target objects. Semantic SLAM combines object semantic information with visual SLAM to build a semantic map that includes object semantic information, making it easier to achieve more complex interaction tasks. Therefore, this paper combines visual SLAM and semantic information extraction algorithms to implement semantic SLAM for the visually impaired assistance system.

- ORB-SLAM2

The feature point-based visual SLAM algorithm is used in this paper to implement semantic SLAM. ORB-SLAM2 is one of the most widely used and high-performance algorithms in feature point-based methods. This algorithm can be used for monocular, stereo, and RGB-D visual SLAM and has the functions of relocalization and loop closure detection. This paper uses ORB-SLAM2 as the basic framework to implement semantic SLAM required for the visual assistance system, which not only constructs a sparse map of the scene and locates the hands of visually impaired individuals but also builds a semantic map that includes semantic information of target objects, i.e., to obtain the location information of target objects.

- YOLO V5s

The semantic SLAM algorithm designed in this paper requires the construction of a 3D semantic map of the scene, which involves the segmentation of 3D point clouds of target objects to label semantic information such as their category and position in the scene. Although the two-stage methods of Faster R-CNN series algorithms have high accuracy, they are difficult to use in real-time detection scenarios with multiple steps. One-stage object detection algorithms based on deep learning have shown great advantages in detection performance, especially the YOLO series algorithms, which are widely recognized and applied in the field of object detection because of their fast and accurate detection performance [17,18,19]. Therefore, this paper selects YOLO V5 based on convolutional neural networks as the algorithm for extracting the semantic information of target objects.

- Semantic SLAM

**Figure 4:**
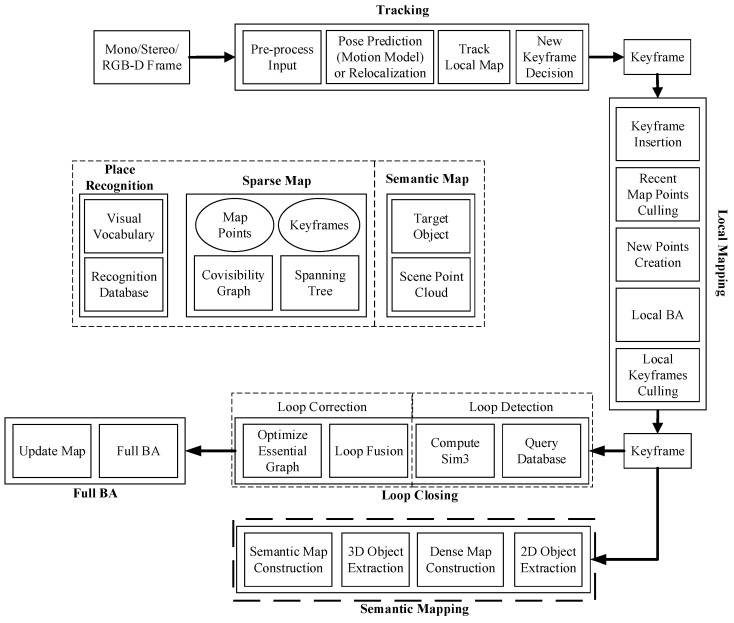
The framework of the semantic visual SLAM.

This paper presents a semantic SLAM framework for the visual perception module of the visually impaired assistive system, as shown in Figure 4. As an improvement to the ORB-SLAM2 algorithm, a semantic mapping thread is added to its original three-thread structure. ORB-SLAM2 is still responsible for real-time pose estimation and sparse map construction. In order to alleviate the burden on the semantic mapping thread, it was added after the local mapping thread. The keyframe needs to be forwarded not only to the loop detection thread but also to the semantic mapping thread for semantic map construction. In the semantic mapping thread, the RGB image corresponding to the current keyframe is first subjected to object detection. Then, a dense map of the scene is constructed using the depth map, and the object detection results are combined to locate all object point clouds in the dense map, thereby extracting the 3D targets in the scene. Finally, the positions, categories, and other semantic information of the 3D targets are annotated on the dense map.

In addition, when a visually impaired person uses the system to obtain a target object, i.e., the system works in the relocalization mode, the camera may lose track because of scene changes or drastic camera movements. In this case, the camera can be relocalized in the scene using ORB-SLAM2’s BoW-based relocalization algorithm. The local mapping thread in ORB-SLAM2 is responsible for constructing the current observed scene map. However, it first needs to determine the redundancy in the current keyframe. If it has high similarity with other keyframes, the current keyframe will not be used to build the map.

## 3. Algorithm Verification

To verify the feasibility and effectiveness of the visually impaired assistance system, we conducted several experimental validations, including the visual SLAM algorithm and semantic information extraction algorithm.

### 3.1. Performance of Object Detection with YOLO V5s

This paper uses the YOLO V5s object detection algorithm to implement the semantic information extraction function of the visual assistance system. In order to evaluate the performance of this algorithm in the visual assistance system, a dataset was created for the experiment, and training and testing were conducted.

#### 3.1.1. Dataset Preparation

**Figure 5:**
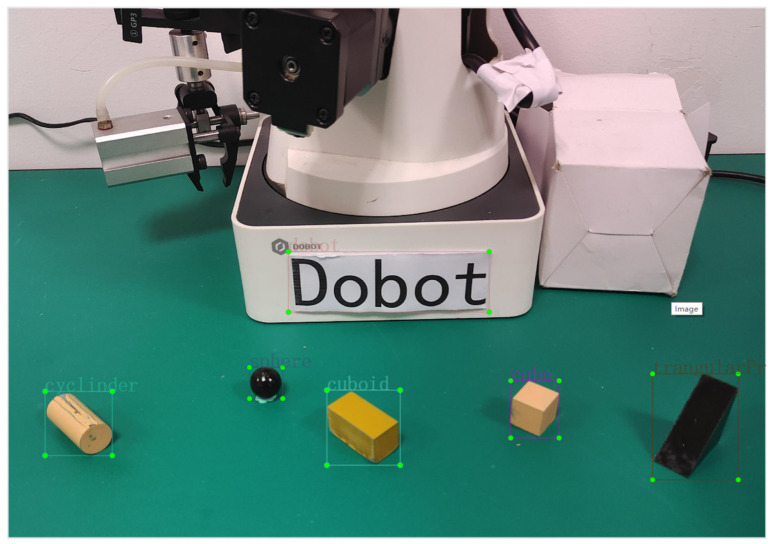
Dataset annotation of five target objects.

Sample datasets were collected by a depth camera, including the following five target objects: a cuboid, a triangular prism, a cylinder, a sphere, a cube, and the Dobot manipulator. Therefore, a category index table was established as {0: “cuboid”, 1: “triangular prism”, 2: “cylinder”, 3: “sphere”, 4: “cube”, 5: “dobot”} and digital indices were used to replace the object category during model training, as shown in Figure 5. The robot gripper’s range for opening and closing is about 27.5 mm. Therefore, the five target objects selected in the experiment could not exceed the size of 27 mm. In Figure 5, the length, width, and height of the cuboid are 25 mm, 15 mm, and 15 mm; the height of the triangular prism is 15 mm, and the hypotenuse is 20 mm; the height of the cylinder is 25 mm, and the diameter of the base circle is 15 mm; the diameter of the sphere is 15 mm; and the edge length of the cube is 15 mm. These specific dimensions were chosen to ensure that the objects were within the optimal range for accurate depth perception by the RGB-D camera while also being suitable for manipulation by the robotic arm.

**Figure 6:**
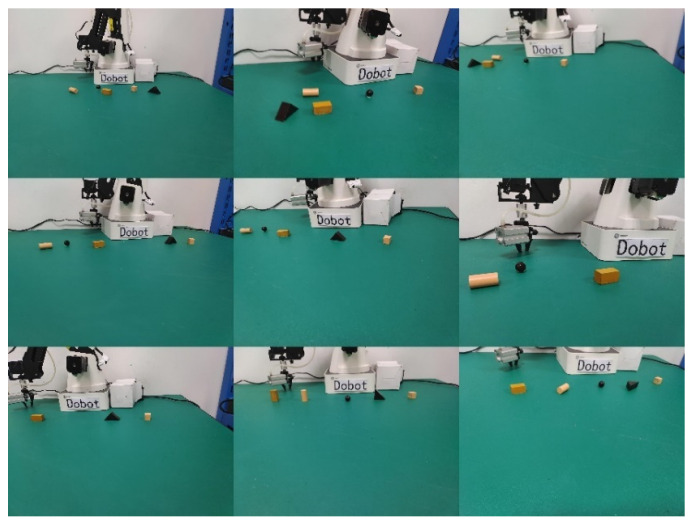
Example of 9 sample pictures for labeling and training.

A total of 435 sample images were collected in the experimental environment, and nine image datasets were collected, as shown in Figure 6. After collecting the dataset, each sample was labeled, including annotating the categories of all targets in the sample and the information of the rectangular bounding box. The LabelImg open-source tool was selected for dataset annotation because of its graphic interaction interface. The labeling format was set to YOLO format, and the labeling results were saved in a corresponding txt file. After labeling, the dataset was divided into training and testing sets in an 8:2 ratio, which was used to estimate the network model parameters and evaluate the network model’s performance during the training process, respectively.

#### 3.1.2. Model Training

Based on the GPU configuration of the training environment for the model built in this paper, the iteration number for training was set to 300, and the batch size was set to 16, which means that 16 samples were selected as the network input for each iteration to improve accuracy and accelerate model training.

**Figure 7:**
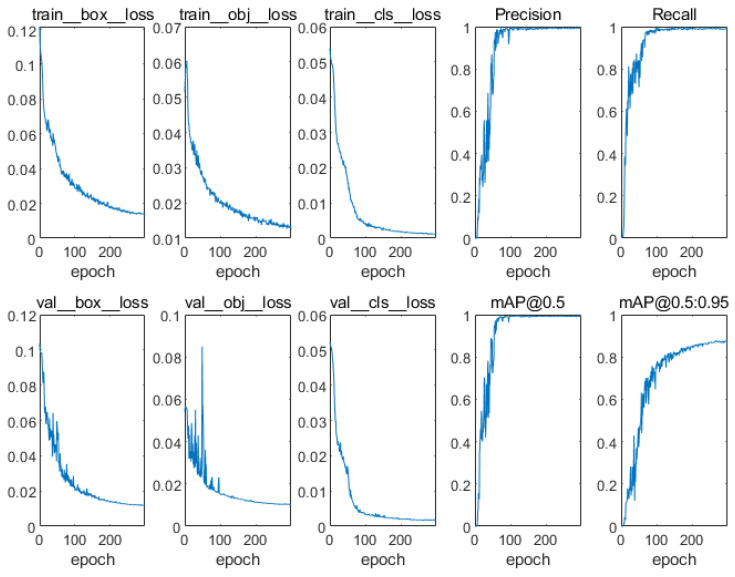
Changes in loss and metrics during training.

After training, the weight file of the YOLO V5s model was obtained. The changes in various losses and indicators during training are shown in Figure 7. It can be seen that with the increase in iteration number, the losses in the training set and test set gradually decrease, while the precision rate *P*, recall rate *R*, and *mAP* gradually increase and eventually converge.

**Figure 8:**
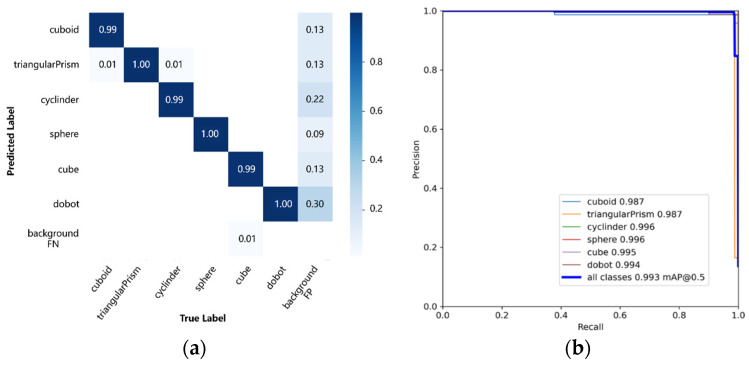
Detection results on test dataset. (**a**) Confusion matrix and (**b**) PR curve.

Meanwhile, the confusion matrix and PR (precision–recall) curve for the detection results of the six types of objects, including the rectangular parallelepiped, triangular prism, cylinder, sphere, cube, and Dobot manipulator, were plotted on the test dataset, as shown in Figure 8. In a small portion of images in the test dataset, the background may be predicted as the target object, but overall, the classification performs well. The PR curve indicates that the YOLO V5s model trained in this study achieves the highest AP_0.5_ of 99.6% and an mAP_0.5_ of 99.3% for all categories, indicating that the model not only has high accuracy but also has high recall and a low probability of missing detections, meeting the requirements of the assistive system for the visually impaired.

#### 3.1.3. Model Deployment and Target Detection

After completing the training of YOLO V5s, the optimal weights saved based on the changes in loss and accuracy were used as the network model configuration and deployed in the visual assistance system to achieve semantic information extraction.

**Figure 9:**
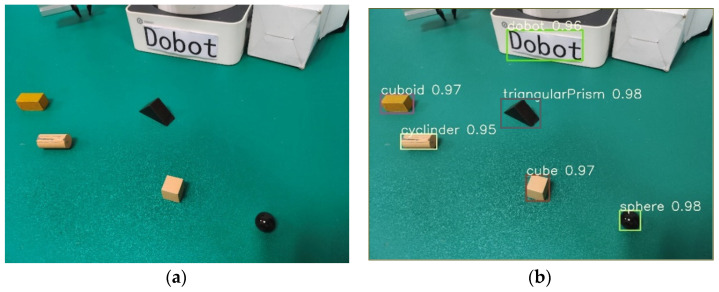
Target detection results after model deployment. (**a**) Original image and (**b**) detection result.

To verify the target detection performance of the deployed model in the experimental environment, the model was tested on randomly collected images. The detection results include the predicted category, confidence, and position information of the target objects, where different objects are distinguished by different colored detection boxes. Figure 9a shows the original detection image, and Figure 9b shows the detection results. The confidence of detecting the Dobot robotic manipulator is 0.96, the cuboid is 0.97, the cylinder is 0.95, the cube is 0.97, the triangular prim is 0.98, and the sphere is 0.98. It can effectively reveal the target objects’ categories, positions, and confidences.

### 3.2. Performance of Semantic SLAM

The visually impaired assistance system based on the semantic visual SLAM designed in this paper has two working modes including SLAM and relocalization. In the SLAM mode, the system can estimate camera motion and construct a sparse map and semantic map of the scene. The coordinates of the map points are obtained by transforming the pose estimation results to the global coordinate system, so the real-time localization effect of the semantic SLAM module of the visual perception module is crucial. In relocalization mode, the system relocalizes the current position based on the sparse map of the scene. After relocalization is finished, the system can enter the normal real-time localization state and execute the grasping operation based on the localization result. Therefore, the localization effect of the system after relocalization directly affects its reliability. This section analyzes the overall localization results of the system in SLAM and relocalization modes and verifies whether the localization effects of both modes meet the system requirements.

#### 3.2.1. Localization Accuracy

**Figure 10:**
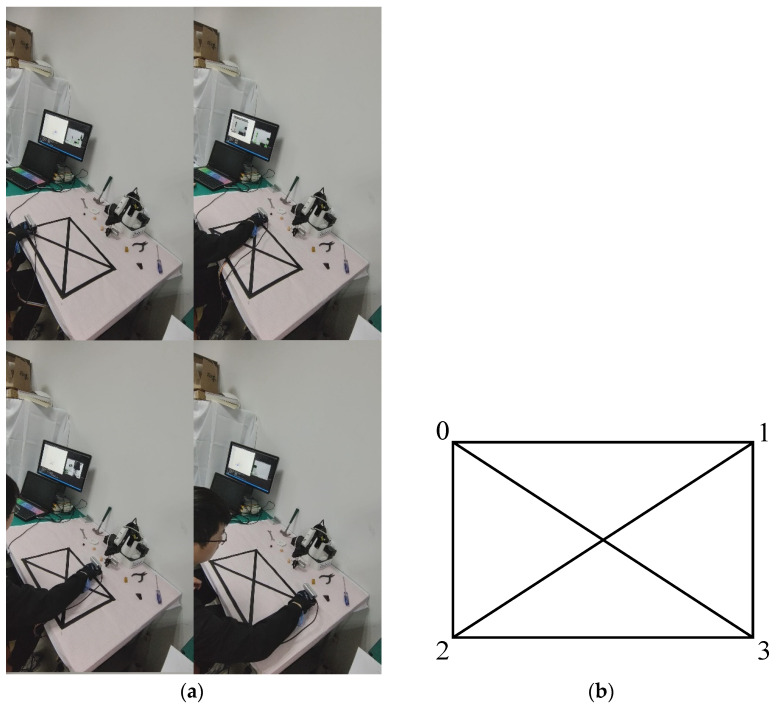
Two-dimensional pattern marked on the coordinate paper. (**a**) Process of collecting motion trajectories. (**b**) Pattern of coordinate paper.

In this section, the positioning accuracy of the system built in the experimental environment is verified to ensure its effectiveness in practical use. The experimental process is shown in Figure 10a, the experimenter wears the glove device and controls their hand to move slowly along the trajectories. Since visually impaired individuals will mainly use the system for operations on a desk, a two-dimensional pattern is marked on the coordinate paper on the desk, as shown in Figure 10b. The pattern is rectangular, with a size of 0.30 m × 0.45 m and four endpoints connected through various paths.

**Figure 11:**
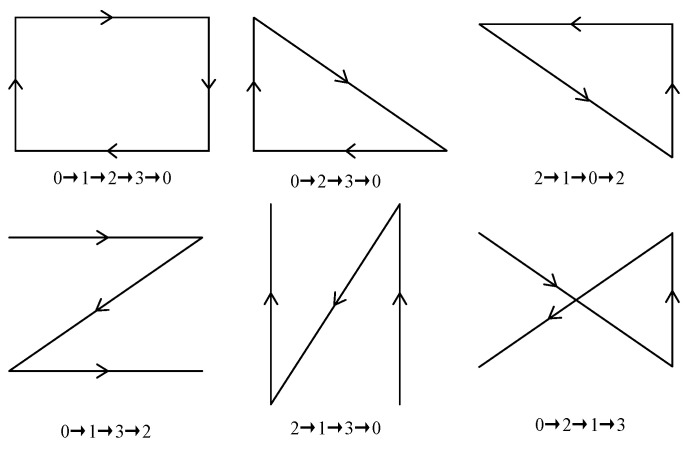
Six different trajectories for determining the accuracy of semantic SLAM camera pose estimation.

Six different paths are set in the experiment, as shown in Figure 11, to represent the ideal camera trajectories. The accuracy of semantic SLAM camera pose estimation can be qualitatively analyzed by judging the overlap between the estimated camera trajectory and the ideal trajectory.

**Figure 12:**
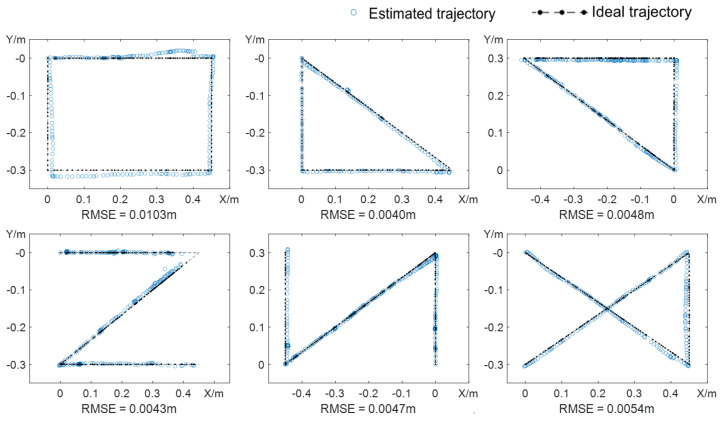
Comparison between the estimated trajectory and ideal trajectory under SLAM mode.

The comparison results with the SLAM mode are shown in Figure 12, where the dotted line represents the estimated trajectory of SLAM and the dashed line represents the ideal trajectory of the different paths arranged on the table. It can be observed that the estimated trajectory generally fits well with the ideal trajectory, indicating that the semantic SLAM algorithm can accurately estimate the position of the camera during movement. However, the error is relatively large at the first turning point of the first “Z”-shaped estimated trajectory because of the high-speed movement and limited computational capabilities, which fails to capture the image frame at the turning point. Therefore, when using the vision assistance system for the visually impaired, the hand movement speed should be controlled within 3 cm/s to prevent tracking failures. Moreover, RMSE (Root Mean Square Error) values for six types of trajectories are calculated to verify the localization accuracy in Figure 12. The maximum RMSE is 0.0103 m for the first trajectory, and the average RMSE across all trajectories is approximately 0.0056 m. The experimental results indicate that the localization accuracy of the depth camera in the SLAM mode can meet the requirements for subsequent interaction and grasping tasks.

#### 3.2.2. Semantic Mapping

**Figure 13:**
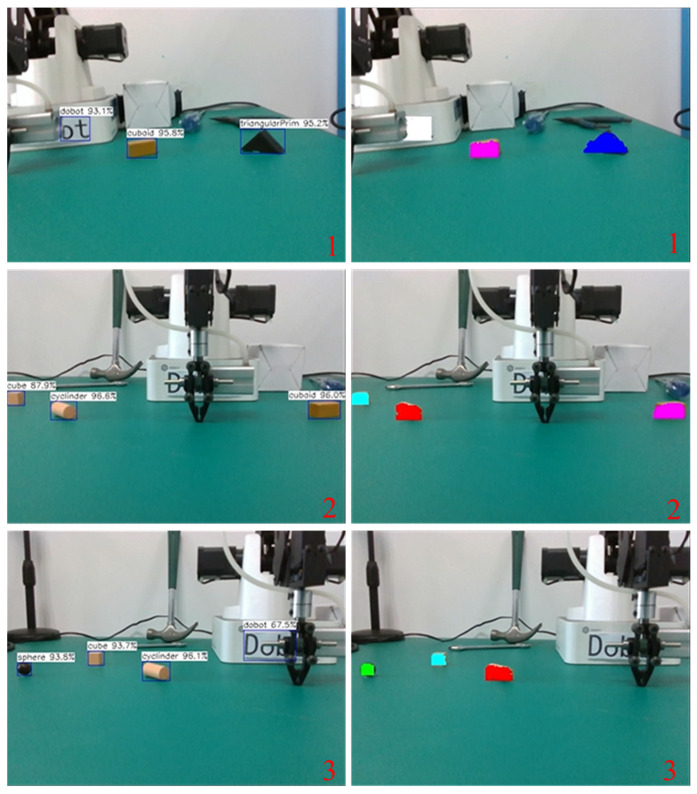
The process of semantic mapping while scanning the targets from right to left as a sequence of 1->2->3.

The semantic SLAM designed in this paper can estimate its own pose and build both a sparse map and a semantic map of the scene. This section tests the accuracy of building the semantic map. The semantic mapping process is shown in Figure 13 while the camera moves from right to left as a sequence of 1->2->3 on the table. The left images show the results of object detection using YOLO V5s, which can detect target objects with high confidence. The right images show the results after filtering out illegal pixels inside the detection box based on the depth values, using different colored pixels to annotate the objects. Here, cyan represents the cube, green represents the sphere, blue represents the triangular prism, white represents the Dobot robotic manipulator, red represents the cylinder, and pink represents the rectangular prism.

**Figure 14:**
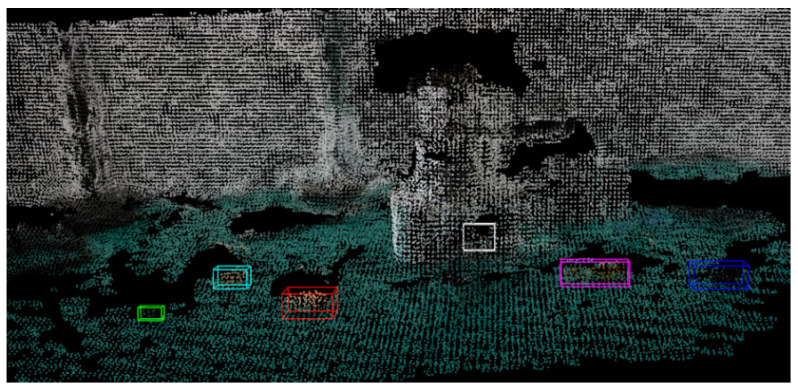
The result of semantic mapping.

By mapping 2D to 3D objects, the final semantic map is built, as shown in Figure 14. The map uses different colored bounding boxes to mark the positions and sizes of the target objects, which can reveal semantic information. This result verifies the feasibility and accuracy of the semantic SLAM algorithm designed in this paper to provide coordinate information for the visually impaired assistive system.

**Figure 15:**
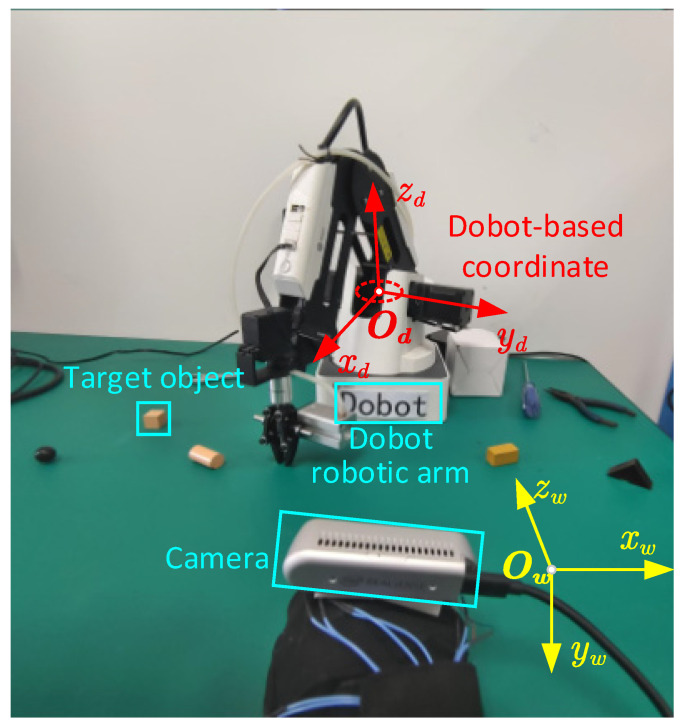
The relationship among the different coordinates.

During camera motion, localization is estimated by the semantic SLAM algorithm, which has been validated for its accuracy in the dataset and experimental environments to meet the requirements of the assistive system design. Next, the accuracy of the semantic map established by the system is tested through experiments. Assuming the target object to be grasped is a cube, its coordinate relationship is shown in Figure 15.

The client receives the global coordinates of the cube, Dobot manipulator, and camera in the semantic map from the map server, denoted as $P_{ow}$, $P_{dw}$, and $P_{cw}$, respectively. They need to be transformed into the Dobot-based coordinate system for grasping. The transformation relationship is given by the following equation:

(1) $$P_{od}=R_{dw}\left(P_{ow}-P_{dw}\right)+t_{bias}$$

where $P_{od}$ is the coordinate of the cube in the Dobot-based coordinate system and $R_{dw}$ is the rotation matrix from the global coordinate system to the Dobot coordinate system. To ensure the accuracy of coordinate transformation, the visually impaired individual should keep their hand in a natural state on the table as much as possible. Thus, we have

(2) $$R_{dw}=\left[\begin{array}{ccc} 0 & 0 & -1\\ 1 & 0 & 0\\ 0 & -1 & 0 \end{array}\right]$$

where $t_{bias}$ is the offset of the Dobot to the origin coordinate system, which can be determined according to its size. Therefore, we have

(3) $$t_{bias}={\left[\begin{array}{ccc} 79 & 0 & -109 \end{array}\right]}^{T}$$

Finally, the Dobot robotic manipulator needs to place the target object directly in front of the visually impaired individual. The camera and hand are in the same position, so we set the object placement point 50 mm in front of the camera. Using the same transformation method as for the target object coordinates, we can obtain the coordinates of the placement point in the Dobot-based coordinate system and place the target object in the correct position.

It should be noted that the map’s global coordinate system is the camera’s posture when the image’s first frame is captured. In order to reduce the influence of camera rotation on positioning accuracy, it is recommended to keep the *z*-axis of the camera coordinate system parallel to the *x_d_*-axis of the Dobot-based coordinate system as much as possible in the initial state.

The visual impairment system is mainly used for tabletop scenarios facing visually impaired people. Therefore, it is only necessary to measure the *X_d_* and *Y_d_* coordinates of the target object and compare them with the positioning results in the semantic map, taking the Dobot-based coordinate system as the reference. The measurement results and the semantic map positioning results are shown in Table 2 below, where the error is described using the Euclidean distance and the error calculation formula is $e=\sqrt{{\left(X_{dm}-X_{dl}\right)}^{2}+{\left(Y_{dm}-Y_{dl}\right)}^{2}}$.

The three-dimensional coordinates of all map points in the semantic map are determined based on the pose estimation results. For a particular target object in the map, its three-dimensional coordinates are obtained by calculating the average of the coordinates of all surface map points. The Dobot robotic manipulator has a repeat positioning accuracy of up to 0.2 mm. When the diameter of the target object ranges from 10 to 27 mm, a centimeter-level positioning accuracy of the target object in the semantic map is required to complete the assisted grasping operation. The average positioning error of the semantic map constructed by the semantic SLAM algorithm designed in this paper is about 0.744 cm, which meets the centimeter-level accuracy requirement.

## 4. The Experiment of Grasping the Target Object

After integrating all the modules, the experiments were carried out in a simulated visual impairment environment by asking the experimenter to a wear blindfold and use the designed visual impairment assistance system to grasp the target object.

### 4.1. Generating Maps

**Figure 16:**
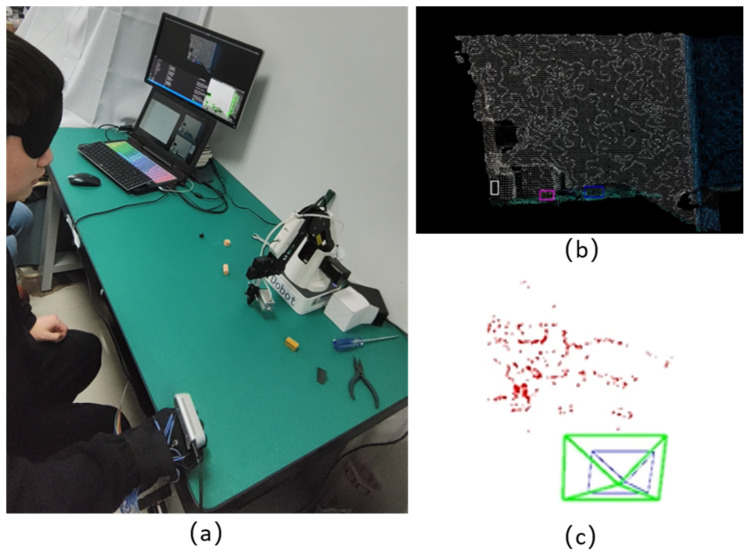
Mapping process status at the start position while scanning the desktop environment. (**a**) Process of the visually impaired person’s operation. (**b**) Generation of semantic map. (**c**) Generation of sparse map.

**Figure 17:**
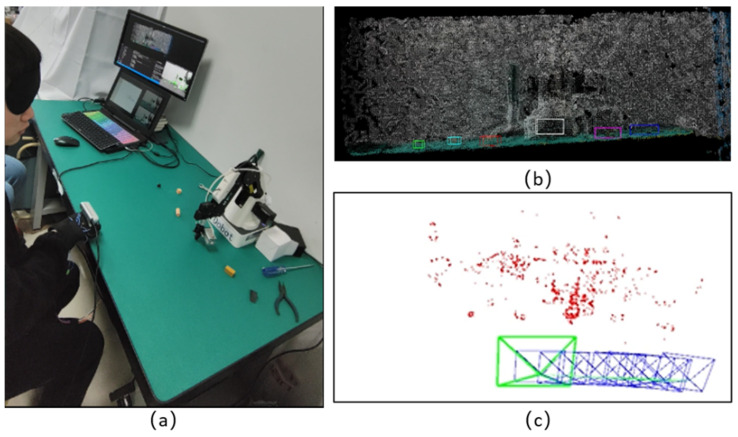
Mapping process status at the end position while scanning the desktop environment. (**a**) Process of the visually impaired person’s operation. (**b**) Generation of semantic map. (**c**) Generation of sparse map.

The system started running from the SLAM mode to generate the map, and the experimenter moved his hand along the edge of the table from right to left while the camera scanned the table. In Figure 16 and Figure 17, (a) shows the process of the visually impaired person’s operation, and (b) and (c) show the semantic map and sparse map generated during the process, respectively. The semantic map marked the size and position of the target object using rectangular boxes in different colors. The sparse map contains 3D map points corresponding to the 2D sparse feature points in the scene and the motion trajectory of the visually impaired person’s hand, where blue and green markings represent the historical and current poses during the movement process. Black map points indicate historical map points the camera cannot observe currently, while red map points indicate the current local map points the camera can observe. When the hand moved to the workspace boundary on the table, the SLAM mode stopped, and the map was saved. The entire mapping process took about 30 s.

### 4.2. Assisted Grasping with a Robotic Manipulator

**Figure 18:**
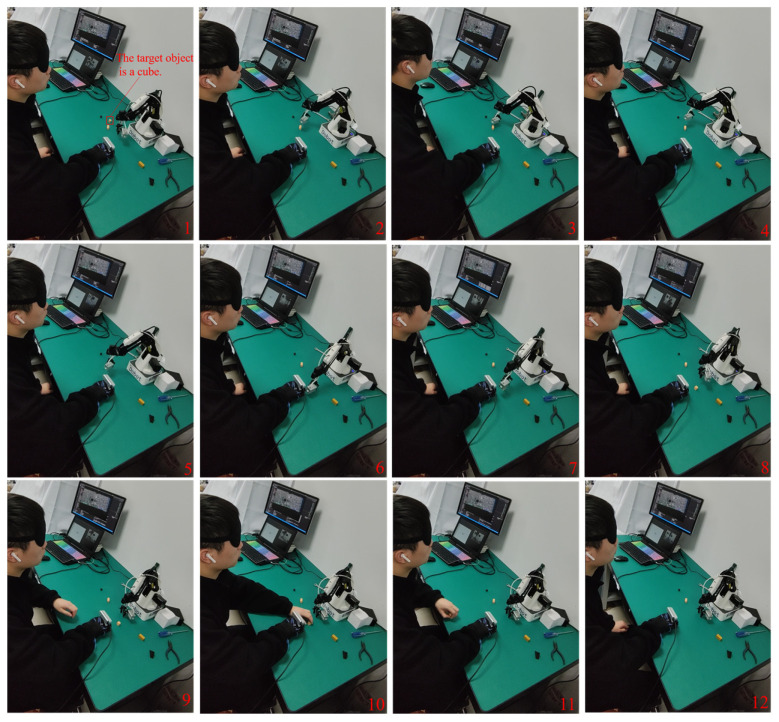
Assisted grasping process with a robotic manipulator as a sequence from 1 to 12.

The robotic manipulator can be used to assist the visually impaired person in retrieving the cube with assisted grasping mode. Firstly, the system recognizes the voice command “grab the cube” from the experimenter, and then it searches for the target object in the generated semantic map. The center of the cube, the Dobot robotic manipulator, and the camera worn on the hand are located, and their global map coordinate information is returned to the client. The process of assisted grabbing is shown in Figure 18, where the Dobot robotic manipulator moves above the cube, performs the grasping, and then places it just in front of the hand for the visually impaired person to obtain. The assisted grasping process with a robotic manipulator takes almost 37 s.

### 4.3. Active Grasping without a Robotic Manipulator

**Figure 19:**
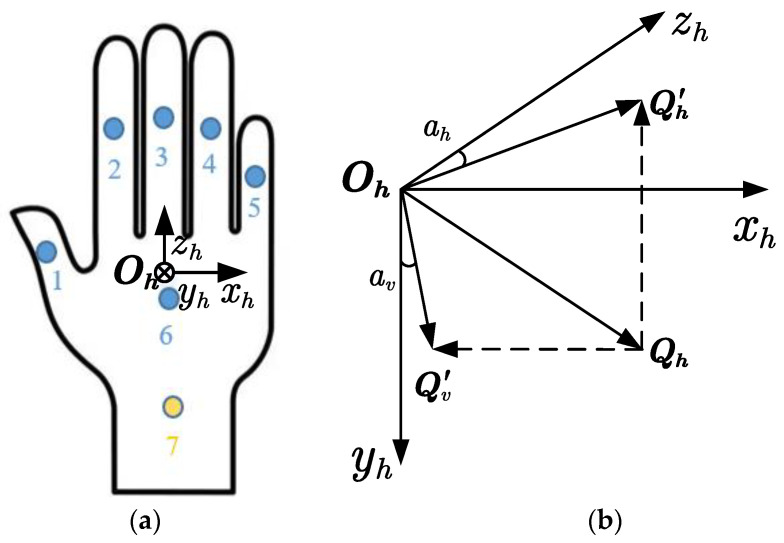
Vibration array design and azimuth calculation. (**a**) The installation positions of the vibration array on the glove. (**b**) Coordinate for the calculation of $a_{h}$ and $a_{v}$.

The visually impaired person can grasp the target object using the vibration feedback array without a robotic manipulator, as shown in Figure 19. Seven vibration motors are installed at different positions on the back of the glove. Assuming the object to be grasped is a cylinder and the hand has been relocated, the client first recognizes the visually impaired person’s voice command “grasp the cylinder” and sends the information on cylinder type to the server. After receiving the command, the server first searches for the target object in the semantic map and then returns the global map coordinates of the cylinder center and the camera on the hand to the client, denoted as $P_{ow}\left(-34.98,11.46,30.33\right)$ and $P_{cw}\left(-23.89,-0.71,11.22\right)$, respectively. The client then transforms the cylinder’s center coordinate to the hand’s camera coordinate system $P_{oc}\left(-11.09,12.17,19.11\right)$.

**Figure 20:**
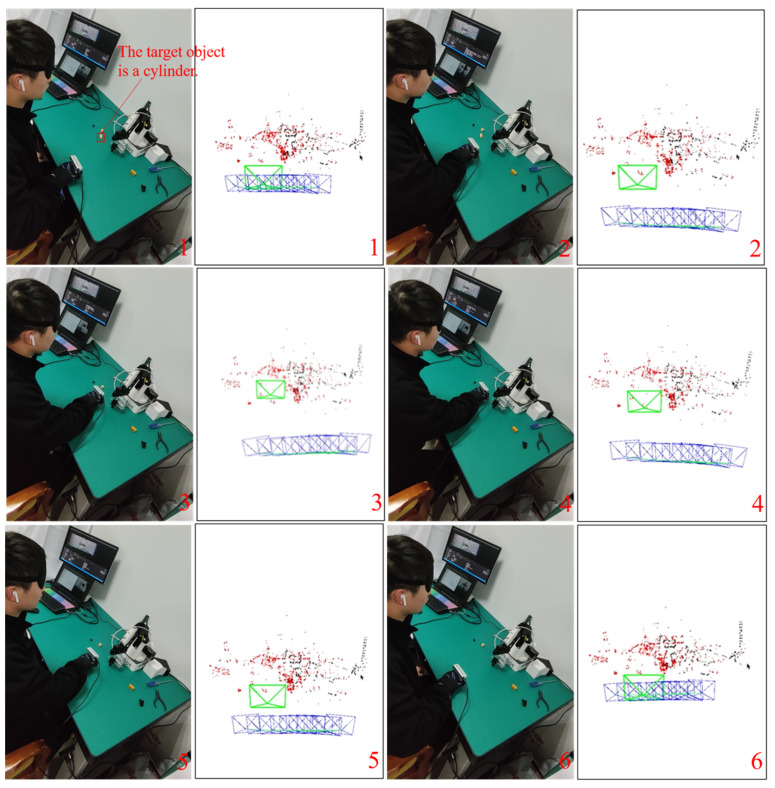
Active grasping process without a robotic manipulator as a sequence from 1 to 6.

The tactile interaction module calculates the horizontal azimuth $a_{h}=-59.87$ degree, vertical azimuth $a_{v}=57.52$ degree, and distance S=25.22 cm of the target object relative to the hand based on this coordinate, which means the target object is in a slightly lower left direction, far from the hand. The experimenter can feel strong vibrations from the second, third, and sixth motors. Based on this vibration information, the experimenter can quickly determine the target’s location and successfully grasp it. Figure 20 shows the sequence of actively grasping the cylinder target and the real-time pose display of the hand-worn camera. The active grasping process takes almost 24 s.

## 5. Conclusions

This paper presents a visually impaired assistive system based on semantic visual SLAM. Semantic visual SLAM technology is applied to assist or guide the visually impaired in completing the task of grasping a target and achieving intelligent interaction tasks in a desktop scenario. In addition, a vibration array feedback device that can convey orientation and distance information is designed as a tactile interaction.

By combining the traditional visual SLAM algorithm (ORB-SLAM2) with the semantic information extraction algorithm (YOLO V5s), real-time camera positioning and map construction can be achieved simultaneously to meet complex and intelligent interaction needs. The visually assistive system designed in this paper is mainly used in a desktop environment. For larger scenes, mobile robots can be used, and navigation algorithms can be designed to achieve more complex intelligent tasks.

## Figures and Tables

**Table 1:** Experimental platform and hardware settings.

Hardware	Model		Parameters
Laptop	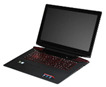	LAPTOP-D3KN7T50	CPU: Intel i5-6300HQ with a clock speed of 2.3 GHz; 16G DDR4 RAM; GPU: GTX 960 M.
Vision sensor	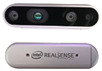	Realsense D435i	Depth image solution: 1280 × 720; RGB image solution: 1920 × 1080; frame rate: 90FPS; depth range: 0.1 m–10 m.
Robotic manipulator	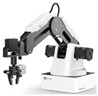	Dobot Magician	Maximum working range: 320 mm; load: 500 g; repeating accuracy: 0.2 mm.
Vibration motor	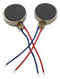	Model 0827	Diameter: 8 mm; thickness: 2.7 mm.

**Table 2:** Localization error of the semantic map (unit: cm).

Category	Measurement Result		Location Result		Error
	*X_dm_*	*Y_dm_*	*X_dl_*	*Y_dl_*	*e*
Cube	−23.6	15.0	−24.1	14.8	0.94
Sphere	−16.3	17.1	−17.0	17.9	1.06
Triangular prism	−10.1	13.1	−10.8	13.0	0.71
Cylinder	10.7	13.1	10.2	13.0	0.51
Cuboid	17.8	13.3	17.4	13.0	0.5

## Data Availability

The data presented in this study are available in this article.
